# Factors Impacting One-year Follow-up Visit Adherence after Bariatric Surgery in West China: A Mixed Methods Study

**DOI:** 10.1007/s11695-024-07227-z

**Published:** 2024-04-15

**Authors:** Jing Liao, Yue Wen, Yiqiong Yin, Yi Qin, Guixiang Zhang

**Affiliations:** Division of Gastrointestinal Surgery, Department of General Surgery, West China Hospital, Sichuan University/West China School of Nursing, Sichuan University, Chengdu, 610041 China

**Keywords:** Adherence, Bariatric surgery, Follow-up, Mixed methods, Obesity

## Abstract

**Purpose:**

Quality follow-up (FU) is crucial after bariatric surgery. However, poor adherence after surgery is prevalent. This research aimed to explore the factors related to FU adherence after bariatric surgery in West China.

**Materials and Methods:**

This study used a sequential explanatory mixed-methods research design. Participants (*n* = 177) were identified from the West China Hospital. Demographic information, disease profile, treatment information, and post-surgery FU information were obtained from the bariatric surgery database of the Division of Gastrointestinal Surgery of the West China Hospital. The survey data were analyzed using logistic regression. Semi-structured interviews with participants (*n* = 10) who had low adherence were conducted. The recording was transcribed verbatim and entered into qualitative data analysis software. Qualitative data were analyzed using a content analysis approach.

**Results:**

Multiple logistic regression revealed that living in Chengdu (OR, 2.308), being employed (OR, 2.532), non-smoking (OR, 2.805), and having less than five years of obesity (OR, 2.480) were positive predictors of FU adherence within one year. Semi-structured interviews suggested that factors related to adherence to FU were lack of motivation, lack of opportunity, insufficient ability, and beliefs regarding consequences.

**Conclusion:**

Factors impacting one-year FU visit adherence after bariatric surgery include not only demographic and disease-related factors but also social and family factors. These results will provide evidence to support healthcare professionals in developing personalized postoperative FU management strategies.

**Graphical Abstract:**

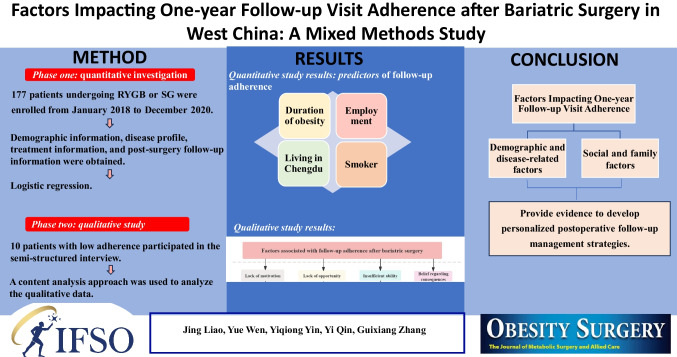

**Supplementary Information:**

The online version contains supplementary material available at 10.1007/s11695-024-07227-z.

## Introduction

Bariatric surgery is a practical approach to treating obesity and obesity-related comorbidities [1–3]. However, some patients may experience weight regain [4], and complications [5, 6]. According to previous studies, the average weight regain was 23.4% of the maximum weight loss after Roux-en-Y gastric bypass (RYGB) [7], the increase in postoperative gastroesophageal reflux disease (GERD) after sleeve gastrectomy (SG) was 19%, and de novo reflux was 23% [8]. The type 2 diabetes (T2D) relapse rate was 20.1% after initial remission [9]. The first year after surgery is crucial because this is usually when most weight loss and postoperative complications occur [10]. Various clinical guidelines have suggested regular postoperative FU visits to guarantee proper care for these patients [11, 12]. However, poor FU compliance is prevalent, with postoperative attrition rates ranging from 3% to over 60%, depending on the setting [13, 14]. As time elapses, the attrition rate also increases. Gourash’s [15] research showed an attrition rate of 14.7% one year after surgery, which increased to 21.8% in the second year after surgery. Poor FU adherence was also related to insufficient weight loss [16, 17], a higher risk of severe nutritional problems [13], and a series of postoperative complications [18].

Many factors could affect adherence to it, including demographic characteristics, disease-related factors, and even distance [15, 19, 20]. However, some factors show inconsistency across various studies. Thereaux and Kedestig demonstrated that women displayed greater adherence to FU visits than men [21, 22]. Meanwhile, another study revealed equal adherence among women and men [19]. In some studies, a higher BMI was associated with improved adherence to FU visits [23]; nonetheless, this connection was not evident in other research [24].

Considering the inconsistency in predictors, the emphasis on quantitative study, and the lack of exploration in the Chinese population, we designed a mixed-methods study to enrich our comprehension of the factors influencing post-bariatric surgery FU compliance.

## Methods

### Study Design

We used a sequential explanatory mixed-methods research design, which offers unique advantages in adherence studies. Thus, we chose this design to increase the depth and scope of the study. In the first phase, we investigated the effects of relevant demographic and disease-related characteristics on FU adherence in bariatric patients using quantitative investigation. Subsequently, in the second phase, we explored personal, family, and social factors influencing FU adherence through semi-structured interviews.

### Phase One—Data Source and Study Population

Patients were identified from the bariatric surgery database within the division of Gastrointestinal Surgery of the West China Hospital, which contains information regarding demographics, medical history, treatments, and FU records of patients who underwent bariatric surgery at the hospital since 2018. Demographic information includes sex, ethnicity, level of education, employment, home address, marital status, and history of drinking and smoking. The disease profile includes the duration of obesity, mental illness, obesity-related comorbidities, and medication history. Treatment information includes operative details and the length of the hospitalization. The FU information includes BMI, surgical outcomes, and remission of comorbidities, among other parameters. In the first year following surgery, four FU appointments were scheduled, including the 1st month, the 3rd month, the 6th month, and the 12th month. Adherence to FU was defined as attending 3 or 4 of these appointments, as established in a previous study [25]. The inclusion criteria for participants in this research were as follows: (a) individuals with Chinese nationality; (b) those who underwent RYGB or SG; (c) those who underwent surgery after January 2018; and (d) individuals with a minimum of one year elapsed since their surgery. Patients who had undergone revisional procedures were excluded. The database recorded 181 patients who were one-year post-surgery from January 2018 to December 2020. Ultimately, a total of 177 participants were enrolled after excluding two patients who underwent revisional procedures and two patients who couldn’t communicate in Mandarin.

### Phase One—Data Collection and Analysis

Data were extracted from the register database. Demographic, disease, and treatment information were sourced from preoperative records. Patients were considered to have participated in FU appointments if they had a registration record and had completed the corresponding examinations (e.g., blood biochemistry, oral glucose tolerance test, etc.). Statistical analysis was performed using SPSS 25.0. Categorical variables were described as numbers and percentages. A *P* value of < 0.05 was considered statistically significant. Logistic regression models were used to examine the association between predictors and FU. To minimize the risk of overfitting the regression model, single-factor logistic regressions were used to select the predictors of FU. Finally, multiple-factor logistic regression was performed on the indicators that had demonstrated statistical significance in the previous single-factor logistic regression.

### Phase Two

In the second phase, we performed descriptive research using semi-structured interviews. The second phase of this study aimed to further explore the individual, social, and family factors that affect the attendance of FU appointments after bariatric surgery, which were not captured in the first phase. The interview guide was developed and revised based on a literature review of bariatric surgery FU and a group discussion. The final interview guide included three questions: “What were the doctor’s recommendations regarding FU after bariatric surgery?” “Why did you not attend the FU visit?” “How did you manage the challenges you encountered after surgery?”.

### Phase Two—Sampling and Recruitment

We employed purposive sampling strategies to select interviewees from among the participants in the quantitative study who exhibited low FU adherence. The sample size was based on data saturation, that is, no additional new themes or codes were identified during the interview [26]. After interviewing and analyzing data from 10 participants, the research team agreed that the codebook had reached saturation, and consequently, data collection was concluded. The first author (L.J.) and another investigator (Q.Y.) identified participants with low FU adherence from the Phase I quantitative study. Subsequently, these individuals were contacted to explain the study’s purpose and methodology and build rapport with them. Finally, participants with a strong willingness to communicate were invited to participate in the interview phase.

### Phase Two -Data Collection and Analysis

Semi-structured interviews were conducted through the telephone at the participant’s preferred time. All interviews were conducted by the first author, a case manager with extensive experience in bariatric surgery management. No repeat interviews were conducted. All interviews were audio-recorded and transcribed verbatim. The mean duration of the interviews was 35 min.

A content analysis approach was used to analyze the qualitative data [27]. NVivo 11 (QSR International) software was used to encode all interview transcripts. After the interviews, two researchers independently listened to the recordings repeatedly and transcribed them within 24 h. The first author read the interview transcription in-depth, extracted the significant content, and then developed the initial codebook. Codes with similar content formed categories and related categories were organized into more comprehensive themes. The survey was conducted in Mandarin or Sichuan dialects. The quotes, codes, and themes were translated into English and back-translated by the investigator, who was skilled in Mandarin, Sichuan dialect, and English.

### Integration of Data and Emergent Themes

First, qualitative and quantitative research results were presented separately, and then data were integrated into the discussion to fully understand the factors influencing patients’ adherence to FU appointments following bariatric surgery. We identified similarities and inconsistencies between the two studies and explored potential reasons for any differences observed. Our study prioritized qualitative results because they explained and complemented quantitative findings in depth [28].

### Ethical Considerations

Ethics approval for the quantitative and qualitative studies was obtained from the Ethics Committee of the West China Hospital of Sichuan University (approval number: 2022506). Before starting the interview, participants gave electronic informed consent. Participants were allowed to refuse to answer any questions or withdraw from the study at any time, with no penalty incurred.

## Results

### Phase One

#### Basic Characteristics of the Study Population and FU Rate

A total of 177 individuals were included in the analysis. Among them, 80.79% of the participants were younger than 40 years old, and 63.28% were female. Most (81.36%) participants had a college degree. Subjects were predominantly employed (65.54%). Further, the majority of the study population (51.98%) was from Chengdu. Among the 177 subjects, only 7.34% were minorities. The proportions of participants with obesity-related comorbidities were as follows: hypertension (19.21%), diabetes (40.68%), hyperuricemia (9.03%), and GERD (15.82%). The proportion of current smokers and drinkers was 26.55% and 12.43%, respectively. The duration of obesity for most participants (60.45%) was over 5 years. Most patients (75.14%) were hospitalized for less than five days. The FU rates for the first, third, sixth months, and one year were 62.71%, 44.07%, 41.24%, and 31.07%, respectively.

#### Predictors of FU Adherence within One year after Bariatric Surgery

In univariate logistic regressions, age, marital status, gender, education, ethnicity, length of stay, drinking alcohol, procedure, hyperuricemia, diabetes, polycystic ovarian syndrome, hypertension, and GERD did not significantly predict FU adherence within one year. Therefore, the above factors were not included in the multiple regression model (Table 1). Multiple logistic regression (Table 2) revealed that patients who lived in Chengdu had better adherence (OR, 2.308; 95% CI, 1.055–5.049). Employed participants had 2.532 times the odds of FU adherence compared to their unemployed counterparts (95% CI, 1.053–6.089). Furthermore, individuals who did not smoke demonstrated 2.805 times higher odds of adhering to FU (95% CI, 1.035–7.601). Lastly, individuals with a history of obesity of less than five years exhibited an increased likelihood of adhering to FU (OR, 2.480; 95% CI, 1.183–5.199).

**Table 1:** The results of a univariate logistic regression analysis of one-year follow-up adherence after bariatric surgery

Factors	Overall (*n* = 177)	Adherence(*n* = 50)	Non-adherence(*n* = 127)	OR	95% CI	*P* value
Age(years)						
18–29	76(42.94)	28(56.00)	48(37.79)	0.290	0.663–3.958	1.620
30–39	67(37.85)	13(26.00)	54(42.52)	0.418	0.253–1.770	0.669
≥ 40	34(19.21)	9(18.00)	25(19.69)			
Marital status						
Married	97(54.80)	23(46.00)	74(58.27)	0.610	0.316–1.179	0.141
Unmarried	80(45.20)	27(54.00)	53(41.73)			
Gender						
Female	112(63.28)	31(62.00)	81(63.78)	0.927	0.471–1.822	0.825
Male	65(36.72)	19(38.00)	46(36.22)			
Education						
College or above	144(81.36)	41(82.00)	103(81.10)	1.061	0.455–2.477	0.890
Senior high school or below	33(18.64)	9(18.00)	24(18.90)			
Ethnicity						
Han nationality	164(92.66)	48(96.00)	116(91.34)	2.276	0.486–10.656	0.296
Minority	13(7.34)	2(4.00)	11(8.66)			
Living in Chengdu						
Yes	92(51.98)	37(74.00)	55(43.31)	3.726	1.808–7.676	0.000
No	85(48.02)	13(26.00)	72(56.69)			
Employment						
Employed	116 (65.54)	41(82.00)	75(59.06)	3.159	1.414–7.054	0.005
Unemployed	61 (34.46)	9(18.00)	52(40.94)			
Duration of obesity						
< 5 years	70(39.55)	29(58.00)	41(32.28)	2.897	1.477–5.681	0.002
≥ 5 year	107(60.45)	21(42.00)	86(67.72)			
Length of stay						
≥ 5 days	44(24.86)	15(30.00)	29(22.83)	1.448	0.696–3.015	0.322
< 5 days	133(75.14)	35(70.00)	98(77.17)			
Procedure						
RYGB	13(7.34)	5(10.00)	8(6.30)	1.653	0.514–5.319	0.339
SG	164(92.66)	45(90.00)	119(93.7)			
Smoking						
No	130(73.45)	44(88.00)	86(67.72)	3.496	1.379–8.866	0.008
Yes	47(26.55)	6(12.00)	41(32.28)			
Drinking alcohol						
Yes	22(12.43)	4(8.00)	18(14.17)	0.527	0.169–1.641	0.269
No	155(87.57)	46(92.00)	109(85.83)			
Mental illness						
Yes	24(12.99)	13(24.00)	11(8.66)	3.705	1.531–8.970	0.004
No	153(87.01)	37(76.00)	116(91.34)			
Hypertension						
Yes	34(19.21)	8(16.00)	26(20.47)	0.740	0.310–1.767	0.498
No	143(80.79)	42(84.00)	101(79.53)			
Diabetes						
Yes	72(40.68)	22(44.00)	50(39.37)	1.210	0.624–2.346	0.573
No	105(59.32)	28(56.00)	77(60.63)			
PCOS (only females)						
Yes	19(16.96)	4(12.90)	15(18.52)	0.625	0.198–2.143	0.481
No	93(83.04)	27(87.10)	66(81.48)			
Hyperuricemia						
Yes	16(9.04)	6(12.00)	10(7.87)	1.595	0.547–4.651	0.392
No	161(90.96)	44(88.00)	117(92.13)			
GERD						
Yes	28(15.82)	10(20.00)	18(14.17)	1.514	0.645–3.555	0.341
No	149(84.18)	40(80.00)	109(85.83)			

*GERD* gastroesophageal reflux disease, *PCOS* polycystic ovarian syndrome, *SG* sleeve gastrectomy, *RYGB* Roux-en-Y gastric bypass

**Table 2:** The results of a multivariate logistic regression analysis of one-year follow-up adherence after bariatric surgery

Factors	B	SE	Wald χ	*P* value	OR	95% CI
Duration of obesity	0.908	0.378	5.781	0.016	2.480	1.183–5.199
Employment	0.929	0.448	4.305	0.038	2.532	1.053–6.089
Living in Chengdu	0.836	0.399	4.388	0.036	2.308	1.055–5.049
Mental illness	0.941	0.503	3.497	0.061	2.562	0.956–6.869
Smoker	1.031	0.509	4.112	0.043	2.805	1.035–7.601

#### Phase Two

**Fig. 1:**
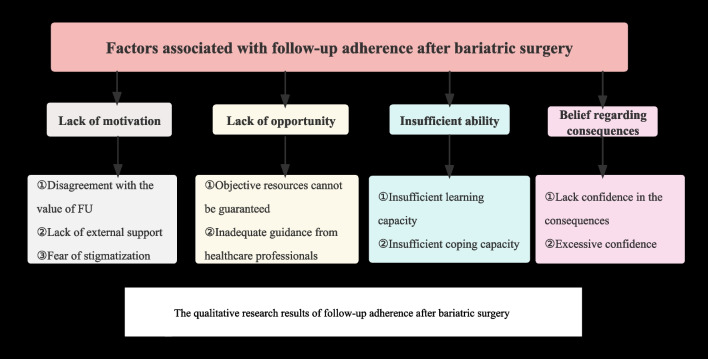
The qualitative research results of follow-up adherence after bariatric surgery

Twelve participants from the quantitative study who had low FU adherence were interviewed. Two subjects declined the interview due to a lack of interest. The demographic characteristics of the participants are shown in Supplementary Table 1. Four themes emerged from the results (Fig. 1): (1) lack of motivation; (2) lack of opportunity; (3) insufficient ability; (4) beliefs regarding consequences. The detailed quotes are shown in Supplementary Table 2.

#### Theme 1: Lack of Motivation

(i) Disagreement with the Value of FU
  As obesity-related co-morbidities improved and there were no postoperative complications, they considered the FU to be less important and gave it lower priority compared to other issues. As participants said, “*I felt very good after surgery without any complications, so I did not need to seek help from healthcare professionals.*”* (Participant 1).*
(ii) Lack of External Support
  The family is an indispensable support provider for patients, providing both material and emotional support. Peers with similar experiences can share information, emotions, and experiences. The patient’s FU attendance may also decrease if they do not receive external assistance from family and peers. “*I wanted to attend FU visits. My brother who also had bariatric surgery at this hospital told me not to follow up.” (Participant 4).*
(iii) Fear of Stigmatization
  Some people believe that bariatric surgery is an “easier” method of weight loss. Thus, doing things the "easy way" was considered "cheating" at weight loss. “*I don’t want my girlfriend to know that I underwent bariatric surgery, so I won’t attend FU appointments. I also left the patient WeChat group recommended by my doctor.*” *(Participant 9).*

#### Theme 2: Lack of Opportunity

(i) Objective Resources Cannot be Guaranteed
  The shortage of medical resources and travel distance can impact patients’ enthusiasm for FU participation. As one participant said,* “On the scheduled FU time, I couldn’t register, and I had no choice but to reluctantly give up on the FU.” (Participant 1).*
(ii) Inadequate Guidance from Healthcare Professionals
  Medical staff need to provide professional guidance on the timing and content of FU; otherwise, patients may not acquire relevant knowledge and may not realize the importance of FU. “*When we were discharged,* the *medical staff told us to follow up, but* they *didn’t remind us when the time came.*” *(Participant 2).*

#### Theme 3: Insufficient Ability

(i) Insufficient Learning Capacity
  It can be difficult for participants with limited capacities to understand the FU information. “*I was illiterate and thought the FU examination was only a gastroscopy.*” *(Participant 1).*
(ii) Insufficient Coping Capacity
  Patients who experience postoperative issues may choose to handle the issue through self-determination based on feelings, excessive dependence on the internet, self-blame, or self-abandonment. “*I have regained weight. I will solve this problem through Baidu and Xiaohongshu.” (Participant 6).*

#### Theme 4: Beliefs Regarding Consequences

(i) Lack of confidence in the consequences: “*Perhaps it is due to genetics, physique, and a sedentary lifestyle that I’ve regained weight. No one can solve my problem.*”* (Participant 10).*
(ii) Excessive confidence: “*I’ve been consistently taking multivitamins, so I shouldn’t experience any nutritional deficiencies.*” *(Participant 4).*

## Discussion

This mixed-methods study provides comprehensive insights into the factors affecting FU adherence following bariatric surgery. The results of the quantitative analysis showed that FU adherence was higher among participants who resided in Chengdu, were employed, did not smoke, and had suffered from obesity for less than five years. Distance was also a factor influencing FU, according to both our quantitative and qualitative investigations. This aligns with the findings of other studies [20, 29]. This relationship may be attributed to increased costs, both in terms of money and time. E-health technologies such as telemedicine, web-based media, smartphone apps, and wearables can help overcome this barrier [30, 31]. Smoking patients were less likely to attend appointments compared to non-smokers. This is consistent with previous studies that identified smoking history as being associated with higher attrition rates at both 30-day [19] and 2-year FU after bariatric surgery [22]. Smokers have self-reported less self-control compared to non-smokers, which may make it challenging for them to follow postoperative instructions [32].

Our qualitative interviews indicated that lack of motivation was the major reason for missing FU appointments. They disagreed with the value of FU and gave it a lower priority than other issues. This aligns with previous studies, which indicated that patients felt a return to normalcy [33–35]. During the "honeymoon period," patients often focus solely on the benefits of bariatric surgery and may not be aware of the potential risks of complications [36].

The patient’s FU attendance may also decrease if they don’t receive external assistance from family and peers. A study on psychological care after bariatric surgery indicated that participants desired the encouragement of their loved ones during the weight loss journey [37]. However, a partner or family member may not fully comprehend the patient’s postoperative experience [37]. Consequently, perioperative counseling should involve the active participation of partners to ensure they understand the changes the patient is undergoing and to encourage and oversee FU appointments.

Our research showed that professional guidance and access to objective resources influenced patients’ attendance at FU appointments, aligning with previous qualitative findings. According to Jumbe’s research, patients experience a cliff from substantial support before the procedure to a sense of abandonment afterward [38]. Few medical staff members proactively remind patients to attend FU, as patients described, "We are old news and old records as surgery goes on [34]". Therefore, healthcare professionals should employ a multifaceted approach to inform patients about the FU. One participant highlighted, "Registration is difficult, and the waiting times for examinations are too long." Online hospitals can effectively alleviate the challenge of securing medical appointments [39].

Insufficient coping capacity, such as self-abandonment, also resulted in patient attrition from FU appointments. This aligns with the previous research, which showed that weight regain often leads patients to feel disappointed and perceive failure, contributing to their reluctance to engage with healthcare professionals [40]. In our study, the participant also mentioned, "No one can solve my weight regain problem." Our interviews indicated that when some patients face problems, their first instinct is to search for solutions on Baidu, which is one of the most commonly used internet search engines in China. Indeed, Breuing’s research indicated that the greatest challenge in providing information to post-bariatric surgery patients comes from the Internet [41]. The internet has emerged as a significant provider of healthcare information [42], influencing the health attitudes and behaviors of a substantial portion of the population [43]. Nevertheless, the quality of online health information exhibits considerable variability [41]. In light of patients resorting to internet-based self-diagnosis and self-disengagement as alternatives to FU care, healthcare professionals should facilitate the establishment of accurate cognitive frameworks.

## Limitations and Future Studies

This study effectively integrated the strengths of both quantitative and qualitative research approaches. Nevertheless, it also exhibits several limitations that warrant attention for future research. Firstly, the qualitative research component involved participants from a single hospital, potentially impacting the generalizability of the findings. Future studies should consider including participants from different institutions to enhance the applicability of the results to a broader population. Additionally, only patients with low FU visit compliance were selected for qualitative interviews, thus focusing solely on barriers to FU. Subsequent research could choose patients with good FU compliance to explore facilitators of FU, thereby overcoming barriers and positively motivating patients to engage in FU.

## Conclusion

This study identified factors impacting one-year FU visit adherence after bariatric surgery. The identified factors include not only demographic and disease-related factors but also social and family factors. Semi-structured interviews suggested that factors related to adherence to FU were lack of motivation, lack of opportunity, insufficient ability, and beliefs regarding consequences. These findings will provide evidence to support healthcare professionals in developing personalized postoperative FU management strategies.

### Supplementary Information

Below is the link to the electronic supplementary material.

Supplementary file1 (DOC 39 KB)

Supplementary file2 (DOCX 17 KB)

## Data Availability

The datasets generated and analyzed during the current study are not publicly available due privacy and ethical reasons, but are available from the corresponding author on reasonable request.
