# Supplementary material for: Factors Impacting One-year Follow-up Visit Adherence after Bariatric Surgery in West China: A Mixed Methods Study
Source: Obes Surg. 2024 Apr 15;34(6):2130–8. doi: 10.1007/s11695-024-07227-z (PMC11127808; doi:10.1007/s11695-024-07227-z)
Supplement: Supplementary file 1 — Supplementary file1 (DOC 39 KB) [file 11695_2024_7227_MOESM1_ESM.doc]

| Participant | Age | Gender | Marital status | Education | Ethnicity |
| --- | --- | --- | --- | --- | --- |
| Patient1 | 47 | Female | Married | Senior high school or below | Minority |
| Patient2 | 27 | Male | Unmarried | College or above | Han nationality |
| Patient3 | 25 | Female | Married | Senior high school or below | Han nationality |
| Patient4 | 38 | Female | Married | College or above | Han nationality |
| Patient5 | 25 | Female | Married | College or above | Han nationality |
| Patient6 | 47 | Female | Married | College or above | Han nationality |
| Patient7 | 28 | Male | Unmarried | College or above | Han nationality |
| Patient8 | 28 | Male | Married | College or above | Han nationality |
| Patient9 | 20 | Female | Unmarried | College or above | Han nationality |
| Patient10 | 46 | Female | Married | Senior high school or below | Han nationality |

**Supplementary Table 1** Demographic characteristics of interviewees(n=10)
