# Supplementary material for: Factors Impacting One-year Follow-up Visit Adherence after Bariatric Surgery in West China: A Mixed Methods Study
Source: Obes Surg. 2024 Apr 15;34(6):2130–8. doi: 10.1007/s11695-024-07227-z (PMC11127808; doi:10.1007/s11695-024-07227-z)
Supplement: Supplementary file 2 — Supplementary file2 (DOCX 17 KB) [file 11695_2024_7227_MOESM2_ESM.docx]

**Supplementary Table 2** Themes, sub-themes, and sample interview quotes

| Themes | Subthemes | Quotes |
| --- | --- | --- |
| Lack of motivation | Disagreement with the value of FU | *My fatty liver has gone from severe to mild. There's no need for further FU visits. (Participant 7)* |
|  |  | *My blood pressure has returned to normal. (Participant 10)* |
|  |  | *I felt very good after surgery without any complications, so I did not need to seek help from healthcare professionals. (Participant 1)* |
|  |  | *I'm really busy, I didn't remember FU at all. (Participant 2)* |
|  | Lack of external support | *I work in the hospital and manage my whole family's health, so my family will not care if I follow up on time after surgery. (Participant 10)* |
|  |  | *I wanted to attend FU visits. My brother who also had bariatric surgery at this hospital told me not to follow up. (Participant 4)* |
|  | Fear of stigmatization | *No one in my family knew I had bariatric surgery. If I visit the hospital frequently, they will start to suspect me. (Participant 3).* |
|  |  | *I don't want my girlfriend to know that I underwent bariatric surgery, so I won't attend FU appointments, I also left the patient WeChat group recommended by my doctor. (Participant 9)* |
| Lack of opportunity | Objective resources cannot be guaranteed | *On the scheduled FU time, I couldn't register, and I had no choice but to reluctantly give up on the FU. (Participant 1)* |
|  |  | *I waited for four hours for a CT last time. It was my turn, but emergency cases were prioritized ahead of me. Every FU visit takes several days, affecting both my work and studies. (Participant 5)* |
|  |  | *I have been to Urumqi, and Xinjiang is indeed too far from Chengdu. (Participant 6)* |
|  | Inadequate guidance from healthcare professionals | *When we were discharged, the medical staff told us to follow up, but they didn't remind us when the time came.” (Participant 2).* |
| Insufficient ability | Insufficient learning capacity | *I was illiterate and thought the FU examination was only a gastroscopy.” (Participant 1).* |
|  | Insufficient coping capacity | *The stomachache must be caused by eating. Severe hair loss must be related to poor rest. I always believed that everything would improve, I thought it was just a matter of time. (Participant 8)* |
|  |  | *I have regained weight. I will solve this problem through Baidu and Xiaohongshu. (Participant 6).* |
| Beliefs regarding consequences | Lack of confidence in the consequences | *Perhaps it is due to genetics, physique, and a sedentary lifestyle that I’ve regained weight. To be honest, I’ve lost confidence, I've been trying to lose weight for decades, and no one can solve my problem. (Participant 10).* |
|  | Excessive confidence | *I've been consistently taking multivitamins, so I shouldn't experience any nutritional deficiencies. (Participant 4).* |
